# Ten Years of Collaborative Progress in the Quest for Orthologs

**DOI:** 10.1093/molbev/msab098

**Published:** 2021-04-02

**Authors:** Benjamin Linard, Ingo Ebersberger, Shawn E McGlynn, Natasha Glover, Tomohiro Mochizuki, Mateus Patricio, Odile Lecompte, Yannis Nevers, Paul D Thomas, Toni Gabaldón, Erik Sonnhammer, Christophe Dessimoz, Ikuo Uchiyama, QFO Consortium, Adrian Altenhoff, Aida Ouangraoua, Alex Warwick Vesztrocy, Benjamin Linard, Christophe Dessimoz, Damian Szklarczyk, Dannie Durand, David Emms, David Moi, David Thybert, Erik Sonnhammer, Evgenia Kriventseva, Haiming Tang, Hirokazu Chiba, Ikuo Uchiyama, Ingo Ebersberger, Jaime Huerta-Cepas, Jesualdo Tomas Fernandez-Breis, Judith A Blake, Leszek Pryszcz, Maria-Jesus Martin, Marina Marcet Houben, Mateus Patricio, Matthieu Muffato, Natasha Glover, Odile Lecompte, Paul D Thomas, Philipp Schiffer, Salvador Capella-Gutierrez, Salvatore Cosentino, Shawn E McGlynn, Shigehiro Kuraku, Sofia Forslund, Steven Kelly, Suzanna Lewis, Tamsin Jones, Tarcisio Mendes de Farias, Taro Maeda, Toni Gabaldon, Wataru Iwasaki, William Pearson, Yan Wang, Yannis Nevers, Yuichiro Hara

**Affiliations:** 1 LIRMM, University of Montpellier, CNRS, Montpellier, France; 2 SPYGEN, Le Bourget-du-Lac, France; 3 Institute of Cell Biology and Neuroscience, Goethe University Frankfurt, Frankfurt, Germany; 4 Senckenberg Biodiversity and Climate Research Centre (S-BIKF), Frankfurt, Germany; 5 LOEWE Center for Translational Biodiversity Genomics (TBG), Frankfurt, Germany; 6 Earth-Life Science Institute, Tokyo Institute of Technology, Meguro, Tokyo, Japan; 7 Blue Marble Space Institute of Science, Seattle, WA, USA; 8 Swiss Institute of Bioinformatics, Lausanne, Switzerland; 9 Center for Integrative Genomics, University of Lausanne, Lausanne, Switzerland; 10 Department of Computational Biology, University of Lausanne, Lausanne, Switzerland; 11 European Molecular Biology Laboratory, European Bioinformatics Institute, Wellcome Genome Campus, Hinxton, Cambridge, United Kingdom; 12 Department of Computer Science, ICube, UMR 7357, University of Strasbourg, CNRS, Fédération de Médecine Translationnelle de Strasbourg, Strasbourg, France; 13 Division of Bioinformatics, Department of Preventive Medicine, University of Southern California, Los Angeles, CA, USA; 14 Barcelona Supercomputing Centre (BCS-CNS), Jordi Girona, Barcelona, Spain; 15 Institute for Research in Biomedicine (IRB), The Barcelona Institute of Science and Technology (BIST), Barcelona, Spain; 16 Institució Catalana de Recerca i Estudis Avançats (ICREA), Barcelona, Spain; 17 Science for Life Laboratory, Department of Biochemistry and Biophysics, Stockholm University, Solna, Sweden; 18 Department of Computer Science, University College London, London, United Kingdom; 19 Department of Genetics, Evolution and Environment, University College London, London, United Kingdom; 20 Department of Theoretical Biology, National Institute for Basic Biology, National Institutes of Natural Sciences, Okazaki, Aichi, Japan

**Keywords:** orthology, viruses, phylogenetic profiling, paralogy, xenology, gene models

## Abstract

Accurate determination of the evolutionary relationships between genes is a foundational challenge in biology. Homology—evolutionary relatedness—is in many cases readily determined based on sequence similarity analysis. By contrast, whether or not two genes directly descended from a common ancestor by a speciation event (orthologs) or duplication event (paralogs) is more challenging, yet provides critical information on the history of a gene. Since 2009, this task has been the focus of the Quest for Orthologs (QFO) Consortium. The sixth QFO meeting took place in Okazaki, Japan in conjunction with the 67th National Institute for Basic Biology conference. Here, we report recent advances, applications, and oncoming challenges that were discussed during the conference. Steady progress has been made toward standardization and scalability of new and existing tools. A feature of the conference was the presentation of a panel of accessible tools for phylogenetic profiling and several developments to bring orthology beyond the gene unit—from domains to networks. This meeting brought into light several challenges to come: leveraging orthology computations to get the most of the incoming avalanche of genomic data, integrating orthology from domain to biological network levels, building better gene models, and adapting orthology approaches to the broad evolutionary and genomic diversity recognized in different forms of life and viruses.

## Introduction

Orthology and paralogy are evolutionary relationships linking homologous genes which diverged via speciation or duplication events, respectively (Fitch 1970). Correctly inferring these relationships is fundamental in many fields of biology. For instance, alignments of orthologous genes are the basis of most systematics and evolutionary studies. Additionally, orthologs tend to conserve their biological functions between different species more than paralogs, as duplications are often followed by functional divergence (Altenhoff et al. 2012; Gabaldón and Koonin 2013; Rogozin et al. 2014). This makes high-quality orthology inference a critical step, with impacts on many downstream analyses ranging from gene functional predictions to biological network interpretation.

Still, predicting orthology from biological sequences remains a challenging problem. New genomes are released every week (Mukherjee et al. 2019) and inferring orthology relationships at least linearly and typically quadratically with the number of available genomes. Thus, a continuous effort of data integration and standardization is essential to keep up with the speed with which novel data arrives. Genomes are shaped by speciation and duplication events, but also gene loss, domain architecture rearrangements and horizontal gene transfers, which complexifies orthology predictions and constantly pushes development of new computational methods (Forslund et al. 2018).

**Fig. 1.**
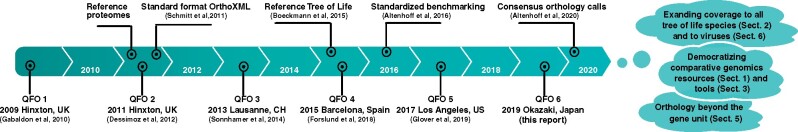
Meetings and key milestones of the past decade. Clouds report ongoing work and the manuscript section (Sect.) where it is discussed.

The Quest for Orthologs (QFO) Consortium was founded in 2009 to address the challenges and opportunities of orthology prediction in the genomic era. This community effort united dozens of researchers interested in orthology database construction, orthology software development, and applications of orthologs (Gabaldón et al. 2009). Ten years later, this consortium has grown into a worldwide community (questfororthologs.org) and biannual meetings are organized to discuss the many aspects of orthology research (fig. 1). This community effort has led to major achievements, including curated reference proteomes for phylogenetically diverse species (Dessimoz et al. 2012), reference species trees useful for orthology predictions (Boeckmann et al. 2015) and an online benchmarking service standardizing the assessment and comparison of orthology inference methods (Altenhoff et al. 2016).

The sixth QFO meeting was organized in conjunction with the 67th National Institute for Basic Biology (NIBB) conference and held in Okazaki (Japan) in August 2019 (www.nibb.ac.jp/conf67, last accessed 15/04/2021), coinciding with the tenth anniversary of the consortium. With 28 speakers from nine different countries, the meeting focused on the latest advances in orthology-related research and treated present and future challenges that may be addressed by this community. Here, we review the main subjects that were addressed during these sessions and survey the future challenges of orthology-related research that will be the focus of the QFO Consortium.

### Orthology Resources: New Tools and Updates

Today, accurate large-scale orthology inference covering hundreds to thousands of genomes remains a challenging task, as demonstrated by the constant development of new orthology inference tools. These methods can be broadly divided into four categories: tree-based, graph-based, hybrid (e.g., graph and tree based) and meta-approaches. We will briefly describe the characteristics that differentiate them (see Altenhoff et al. 2019 for a recent and more extensive review).

Tree-based tools are based on tree reconciliation, in which a gene tree and a reference species tree are compared with assign duplication or speciation events to each internal node of the gene tree. Because they are based on more complex modelizations (evolutionary models) and considered more accurate, tree-based methods are often favored in projects targeting specific clades or involving a limited number (dozens to a few hundreds) of genomes. Their main bottlenecks remain their computational cost (multiple alignments and tree inferences) and the unavoidable assumptions introduced when selecting gene models and delimiting genes into putative gene families prior to the alignments.

Graph-based approaches have been developed to cope with larger sequence volumes. In these tools, the genetic distances accurately modeled in tree-based approaches are approximated via a pairwise distance-matrix built for all studied genes. Then, this matrix is used to build a graph which is decomposed into orthologous gene pairs or groups assuming that orthologous genes are clustered together in this graph (e.g., orthologs are recursively more similar than nonorthologous genes).

Hybrid methods combine both approaches. In a preliminary step they take advantage of the higher scalability of graph-based approaches to infer large sets of putative orthologs which are then refined using tree-based validations.

Finally, meta-methods are combining the output of several methods to refine orthology predictions and increase their robustness. They rely on a variety of combinatorial approaches (see Glover et al. 2019 for a nonexhaustive list) or machine-learning methods (Sutphin et al. 2016).

During the sixth QFO conference, several methods were presented. They mostly belonged to the hybrid and graph-based categories and showed a notable focus on speed and scalability improvement. Wataru Iwasaki presented the software SonicParanoid (Cosentino and Iwasaki 2019), a graph-based approach similar to InParanoid in which sequence similarity scores are not computed by a classical BLAST-based approach but via MMSeqs2 (Steinegger and Söding 2017) to accelerate the distance computation necessary to the graph construction. Steven Kelly presented the second version of OrthoFinder, an hybrid method with a workflow combining graph and tree-based approaches to infer large-scale and accurate orthology and paralogy relationships (Emms and Kelly 2019). A key idea of OrthoFinder was that the scores used to build the graph should account for the gene length bias via BLAST score normalization (Emms and Kelly 2015). In this preliminary step, users can choose between a classical BLAST approach, or one of the faster, less accurate algorithms: DIAMOND (Buchfink et al. 2015) or MMSeq2 (Steinegger and Söding 2017). In the second version, predictions are refined using additional steps based on gene tree reconstruction, species tree root identification, and validation of the detected duplication events. These steps do not require multiple alignment of the sequences of each group but instead use DendroBLAST, a tool to reconstruct approximate phylogenetic dendrograms on the basis of pairwise alignments (Kelly and Maini 2013).

Two other recent tools are SwiftOrtho (Hu and Friedberg 2019) and JustOrthologs (Miller et al. 2019). The former introduced an original approach where inexact k-mer matches via spaced-seeds patterns are combined to a reduced protein alphabet (e.g., amino acids sharing common physicochemical traits are collapsed) to accelerate similarity searches prior to graph reconstruction and MCL (Markov CLustering) algorithm. The latter is a new tool that fits neither a graph-based or tree-based approach and could be related to older orthology inference methods based on the reciprocal best hits idea. JustOrthologs uses alignment-free criteria for defining sequence similarity. It compares genes from two proteomes by 1) looking for at least one, maximum two CDS of identical length and 2) by counting the occurrences of a dinucleotide pair in the exons and dividing it by the total number of dinucleotide pairs in those exons. Orthology inference is then based on a decision tree exploiting these two criteria. Although this approach is fast (no alignments), its usefulness appears restricted to closely related genomes.

The conference was also noteworthy for many major updates that concerned most of the established databases of precomputed orthology relationships. Many talks were related to the latest updates in databases built using graph-based and hybrid methods. Most showed vast expansions in their taxonomic coverages and confirmed their capacity to deal with the current rates of full genome sequencing. Yannis Nevers presented the third version of the OrthoInspector database. This graph-based approach based on pairwise genome comparisons expanded to 4,763 species and its website was redesigned to allow browsing orthology via three subdatabases (*Bacteria*, *Archaea*, *Eukaryotes*) and a cross-domain database dedicated to model species (Nevers et al. 2019). Christophe Dessimoz presented the latest state of OMA (Orthologous MAtrix) (Altenhoff et al. 2018) another graph-based approach which has expanded to 2,200 complete genomes (June 2019) and recently introduced novel tools for data visualization tools and semantic data sharing. Ikuo Uchiyama described the last updates of MBGD (MicroBial Genome Database), an orthology database which focuses on *Bacteria*, *Archaea*, and unicellular *Eukaryotes*. MBGD predictions are based on an updated two-step inference pipeline where a faster but less sensitive large-scale UBLAST search is followed by BLASTP and Smith–Waterman alignments at the genus level (Xiang et al. 2020). Among the richest databases for bacterial data, MBGD has expanded to 6,218 species in 3,566 genera. Similarly, OrthoDB 10, another graph-based method, has expanded to 13,772 species, of which 6,488 are viruses and 5,609 are bacterial genomes (October 2019) (Kriventseva et al. 2019). OrthoDB predictions are based on an updated pipeline where MMseqs2 is used for homology searches and novel heuristics are used for better selection of seeds for the graph construction and filtering of mispredicted gene fusions.

The category of meta-methods was represented by Paul D Thomas that described the latest developments of PANTHER (Protein ANalysis THrough Evolutionary Relationships) version 14. The corresponding database expanded to 142 complete genomes (Mi, Muruganujan, Huang, et al. 2019). This last iteration particularly focused on collaboration with biocurators (Gaudet et al. 2011) and the Ensembl Compara resource (Herrero et al. 2016), leading to refined gene family boundaries for inferring phylogenetic trees and updated annotations (Mi, Muruganujan, Huang, et al. 2019).

Beyond the meeting, it is noteworthy to mention the recent updates of OrthoMaM 10 (Orthologous Mammalian Markers), an expert database focusing on high-quality tree reconciliation in mammals that expanded to 47 complete genomes with a focus on resolving exon positions in CDS alignments to infer fine-grained exon orthology (Scornavacca et al. 2019). Finally, EggNOG 5 (Evolutionary genealogy of genes: Nonsupervised Orthologous Groups), another hybrid method, expanded to 5,090 species including a large expansion in viral genomes (352–2,502, October 2019).

### Scalability, Standardization, and Benchmarking

The long list of orthology database expansions demonstrates the capacity of current orthology inference tools to encompass a large number of genomes, which is particularly true for graph-based and hybrid approaches. Still, the conference highlighted that database updates aiming for exhaustive inclusion of all sequenced genomes may become an unsustainable option in the near future. As shown by the most recent database updates, many authors are now initiating their predictions on very similar, if not identical, gene models and sequence sets. Many tools rely on the Uniprot reference proteomes data set and in many cases, the same pairwise aligners (BLAST, MMseq2, etc.) are used to compute similar sequence distances.

The last iteration of EggNOG was one of the first attempts to introduce new strategies to limit such computation duplicates. Similarly to other hybrid methods, it uses graph-based predictions refined by gene-tree reconciliation and species delineation (Huerta-Cepas et al. 2019). But the distance matrix used to build the graphs was derived from an all-against-all Smith–Waterman matrix provided by the SIMAP (Similarity Matrix of Proteins) project (Arnold et al. 2014). This project proposed to compute similarity metrics between well-established and stable gene models and organize them in a shared database. This approach offers the advantage of avoiding to recompute distances between gene models that remain unchanged between genomes releases, and appears as an alternative strategy that could respond to growing concerns related to the increasing environmental footprint of Big Data computations (Lucivero 2020).

The critical point of genome counts and scalability was also thoroughly discussed by Mateus Patricio via the example of Ensembl Compara (Herrero et al. 2016). At the time of the meeting (Ensembl release 96, 199 genomes), Ensembl Compara had ∼50 different pipelines summing up to around 59.2 million jobs per release and ∼51.8 CPU-years in total, run four times a year. However, the Darwin Tree of Life project (DToL, see www.darwintreeoflife.org, last accessed 15/04/2021) will generate a data deluge of 66,000 high-quality annotated genomes from all eukaryotic species found in the British Isles. Consequently, Ensembl Compara has been enhancing its release capabilities and introduced new ways for quantifying and monitoring database changes between releases. By using new statistics such as Jaccard Index (Jaccard 1901) and Gini Coefficient (Gini 1921) based on gene counts, Ensembl Compara can ensure that the amount of changes the database undergoes between releases falls within the expected range. Another metric that has been heavily used to guide the impact of changes is the Gene Order Conservation (GOC), a score which indicates how many of the four closest neighbors of a gene match between orthologous pairs and are in the same relative order (synteny). Better scalability has also served as motivation to look into innovative ideas such as validating predicted homologies via Deep Learning algorithms. This led to the creation of a Google Summer of Code (GSoC) project (github.com/EnsemblGSOC/compara-deep-learning) to evaluate the feasibility of such an approach. Implementation and large-scale tests are currently ongoing in Ensembl Compara (personal communication).

Another major goal of the QFO Consortium continues to be facilitating comparisons among different methods—emerging and established—and the meeting featured several talks touching on standardization and benchmarking. On benchmarking, Adrian Altenhoff presented improvements of the QFO benchmark service, in particular to address uneven species sampling in some of the phylogeny-based tests. Also related to the service, Salvador Capella-Gutierrez reported on the migration of the service back-end to OpenEBench, ELIXIR’s platform for community benchmarking. These services and outcomes from the discussion sessions are reported in a separate paper dedicated to the QFO benchmark service (Altenhoff et al. 2020).

Progress on standardization was reported in terms of nomenclature, ontologies, and tools to facilitate reuse and interoperability. Tamsin Jones described the aim of the Vertebrate Gene Nomenclature Committee (VGNC) to name genes across selected vertebrate species in a way which is consistent with orthology relationships, while also maintaining biological accuracy, memorability, agreement with the literature, and uniqueness (Braschi et al. 2019). Tarcisio Mendes de Farias presented improvement of the Orthology Ontology (Fernández-Breis et al. 2016; Anon), which now supports Hierarchical Orthologous Groups (HOGs). He also noted semantic differences among current Resource Description Framework (RDF) interfaces among orthology databases, which hampers data exchange and querying (Sima, Dessimoz, et al. 2019). On a related topic, Ana Claudia Sima introduced BioQuery, a system to enable semantic queries across federated bioinformatics databases (Sima, Mendes de Farias, et al. 2019). Lastly, also relevant to standardization are tools for the visualization and analysis of HOGs, for example, sets of genes that are inferred to have descended from a common ancestral gene within a species clade. These tools are implemented in the iHam and pyHam libraries (Train et al. 2019), and are compatible with several well established databases such as Ensembl (Yates et al. 2020) or Hieranoid (Kaduk and Sonnhammer 2017).

### Towards Flexible Phylogenetic Profiling

The sheer number and phylogenetic diversity of the available genome sequences provide an excellent foundation for tracing the evolution of proteins and their functions across species and through time. The presence/absence pattern of orthologs in a phylogenetically ordered species collection is summarized in phylogenetic profiles. For individual proteins, such profiles allow to parsimoniously infer their minimal evolutionary age by assigning them to the last common ancestor of the two most distantly related taxa the protein is present in (this, to the condition that no horizontal transfer is involved). In addition, they inform about lineage-specific retention, loss or duplication during species evolution. In a somewhat different approach with the same goals, Paul D. Thomas presented Ancestral Genomes, a new online resource intended to infer the set of protein coding genes present in the last common ancestral genomes of fully sequenced genomes across the tree of life (Huang et al. 2019). The inferences are made from the comprehensive set of over 15,000 gene trees in the PANTHER resource (Mi, Muruganujan, Ebert, et al. 2019), which include gene duplication and horizontal transfer events, as well as parsimony-based inference of gene loss. Each speciation node in a gene tree corresponds to an ancestral gene. The Ancestral Genomes resource also includes ancestral gene function annotations from the Gene Ontology Phylogenetic Annotation project (Gaudet et al. 2011). Paul D. Thomas presented an example of how the resource can be used to identify and characterize (in terms of gene function) evolutionary periods of genome expansion and contraction in the lineage leading from LUCA to placental mammals.

Under the assumption that functionally linked proteins tend to be either retained or lost in a concerted manner, phylogenetic profiling aims at identifying proteins with correlating profiles by considering measures such as the Jaccard index, Euclidean distance, Pearson correlation coefficient, mutual information either individually or in combination (Niu et al. 2017). There are then ample ways to exploit this information, for example, to create, extend, and phylogenetically stratify protein interaction networks (Pellegrini et al. 1999; Tabach, Golan, et al. 2013; Ebersberger et al. 2014; Nevers et al. 2017), to predict subcellular localizations of proteins (Marcotte et al. 2000; Bayer et al. 2014), or to predict protein function (Eisen and Wu 2002). Phylogenetic profiling can also be used to identify genes potentially involved in a given phenotypic process or trait: given the distribution of the phenotype in a set of species, genes with a similar distribution are likely to be involved in the trait under study. Although the conceptual idea of phylogenetic profiling is straightforward, its implementation faces several challenges. Two questions dominate the stage of data compilation. When can a protein be considered orthologous to others in the profile? And how to cope with the ever-increasing amount of genome sequences whose quality is extremely variable? The stage of data interpretation deals then with the problem to assess when two profiles can be considered similar.

Methods for establishing phylogenetic profiles for a set of proteins from a seed species across a collection of target species typically fall into two categories. Unidirectional approaches utilize rapid search algorithms, for example, BLAST (Altschul et al. 1997) or DIAMOND (Buchfink et al. 2015), to identify sequences displaying a significant local similarity to the seed protein. Any BLAST hit exceeding an ad hoc bit score threshold will then serve as a representation of the seed protein in the target species. The advantages are speed and flexibility. The profiles can be optionally limited to individual proteins of interest, the search complexity scales linearly with the number of species, and it is straightforward to extend existing profiles with data from novel species. However, these advantages come at the cost of a loss in specificity. Unidirectional searches have a high false positive rate (Chen et al. 2007). They run a considerable risk of identifying either (out-)paralogs or just proteins sharing individual domains with the seed as best hit in cases when no ortholog is present. Various ways to normalize, for example, NPP (Tabach, Billi, et al. 2013) and SVD (Psomopoulos et al. 2013), and ranking schemes, for example, DPP (Niu et al. 2017), have therefore been developed to faithfully determine the distance/similarity of such phylogenetic profiles despite the expected false positive rate.

Phylogenetic profiling was one of the main topics of this sixth edition of the meeting. Approaches to infer phylogenetic profiles based on the identification of orthologs have a substantially lower false positive rate (Altenhoff et al. 2016), which increases the resolution of the analysis. Yet, the computational complexity of the ortholog searches, as most algorithms scale exponentially with the number of taxa and sequences, is substantial. It leaves the generation of phylogenetic profiles that make comprehensive use of the currently available genomes to institutions with a dedicated computer infrastructure. However, many orthology databases, for example, OrthoDB, OrthoInspector, EggNOG, OMA provide options to query and/or visualize phylogenetic distribution across thousands of species. In particular, a large panel of tools dedicated to phylogenetic profiling has been introduced in the new version of OrthoInspector (Nevers et al. 2019) presented during the conference. For example, each protein page provides direct access to proteins sharing a similar phylogenetic distribution. A phylogenetic profile search allows users to identify all proteins of a species with a given presence/absence profile and to characterize them using a functional enrichment tool. Alternatively, the GO profiling tool allows the visualization of the evolutionary histories of all proteins related to a GO term.

In parallel, several efforts towards scalability and flexibility of phylogenetic profiling have been reported during the meeting. David Moi presented HogProf (Moi et al. 2020), a scalable approach to generate and compare phylogeny-aware profiles exploiting information about duplication, retention and loss events contained in the OMA HOGs. This approach relies on minhashing techniques to avoid all-against-all profile comparisons, allowing for fast retrieval of similar profiles. New methods facilitating the customization of the taxon sets under comparison have also been reported. Odile Lecompte presented a new approach, BLUR (BLAST Unexpected Ranking) (Defosset et al. 2020) a rapid, proteome-scale approach to analyze the protein conservation of two sister clades in order to detect atypical conservation patterns among homologs or orthologs (http://lbgi.fr/blur/). The proposed approach is based on the analysis of the respective conservation of two groups of closely related species compared with a more distant query species used as a reference. The baseline conservation is established at the proteome-level to detect outliers that may correspond to proteins involved in clade-specific evolutionary adaptations.

Ingo Ebersberger presented fDOG (github.com/BIONF/fDOG), a software package facilitating a targeted ortholog search for individual proteins across large taxon collections in linear time. fDOG is a profile-based ortholog search algorithm (Ebersberger et al. 2009) with the option to compile the training data for pHMM generation iteratively on the fly. fDOG is integrated with an automatic scoring of the pair-wise domain architecture similarities between the seed protein and its orthologs. He demonstrated how orthology-based phylogenetic profiles can be rapidly computed across a custom-compiled taxon collection on the fly and displayed and analyzed with PhyloProfile (Tran et al. 2018). Example applications of fDOG include the assessment of gene set completeness (Simão et al. 2015) removing the necessity to concentrate on single-copy orthologs and increasing the resolution to the domain architecture level and the tracing of the eukaryotic core gene set across the archaeal domain to assess which proteins together with the accompanying functions eukaryotes exclusively share with the *Asgard archaea* (Zaremba-Niedzwiedzka et al. 2017). The similarity scores of the domain architecture comparisons can be used instead of binary presence/absence pattern in phylogenetic profiling analyses.

As can be seen, the new developments presented at the QFO meeting explore a wide range of solutions to facilitate and extend the use of phylogenetic profiling. The democratization of these approaches remains a major challenge. Indeed, phylogenetic profiling is largely underexploited outside the comparative genomics community, despite its valuable contributions to the understanding of evolution and genotype/phenotype relationships. In theory, the potential of the approach will continue to develop as the number and diversity of available proteomes increase, however with severe limitations related to the quality of upcoming proteomes. One of the future efforts of the consortium will undoubtedly be to establish a minimum quality requirement for proteomes, on the basis of a set of independent and complementary indicators.

### Orthology beyond the Gene Unit

It has been noted many times that performing orthology analysis with entire protein sequences will inevitably lead to problems for multi-domain proteins (Sonnhammer et al. 2014). For instance, only one domain out of several may be orthologous to another protein whereas the rest of both proteins are made up of different domains. The other domains may even be orthologous to a third protein, manifesting different evolutionary histories of the domains in a protein. This could happen even if the domain architectures are the same. Despite these issues, most ortholog databases and algorithms ignore them and only consider complete proteins. However, at the sixth QFO meeting, a number of presentations on domain-level orthology analysis show that this topic is actively being researched and that progress has been made.

Erik Sonnhammer presented a framework called Domainoid (Persson et al. 2019) that applies the InParanoid algorithm to domains defined by Pfam in order to identify domain-level orthologs. This pipeline allows detection of discordant domain orthologs, that is, cases where different domains on the same protein have different evolutionary histories. He showed that domain-based orthology inference can reveal many orthologous relationships that are not found by full-length sequence approaches and can therefore be a valuable complement to traditional methods. Subgene elements are also taken into account by BLUR (Defosset et al. 2020) presented by Odile Lecompte. This new tool aims at detecting divergence between two related groups of proteomes at different levels: presence/absence of orthologs predicted by OrthoInspector (Nevers et al. 2019) but also gain/loss or accelerated evolution of protein domains or smaller uncharacterized regions. This multi-level comparison provides a comprehensive view of the genetic basis for species adaptation or specialization. As an example, comparison of ciliated and nonciliated fungal species revealed a network of cilia-enriched genes connecting cases of subgene level divergences and gene losses in nonciliated fungi.

Dannie Durand presented a framework, Notung-DM, that reconstructs multidomain evolution using Wagner parsimony in order to reconcile a domain tree with a gene tree, guided by the species tree (Stolzer et al. 2015). This way, various questions about the evolution of domain architectures can be addressed by identifying events such as domain duplication, insertion, transfer, or deletion. For instance, for an example data set, 21% of the domain architectures were found to have domain insertions (three domains on average).

The MBGD database is constructed using the domain-aware algorithms DomClust (Uchiyama 2006) and DomRefine (Chiba and Uchiyama 2014) that separate orthologous domain clusters based on an ab initio score optimization and refinement procedure. Hirokazu Chiba presented an analysis of proteins in MBGD with domains that belong to different ortholog groups. By connecting ortholog groups when a protein is found in both groups and analyzing the resulting domain fusion network they found that proteins involved in signal transduction and secondary metabolites were particularly prone to domain fusions. Comparing different species indicated that extremophiles had unusually few domain fusions.

Ingo Ebersberger introduced FAS (github.com/BIONF/FAS), an approach to compare domain architectures between pairs of proteins and to score their similarity. Integrated with an ortholog search, FAS scores can be used to screen for lineage-specific changes in the domain architecture of orthologs indicative of a change in function. He showed an application of this approach to the phylogenetic profile of the proteome from *Acinetobacter baumannii*, a nosocomial human pathogen, across >2,500 species. This reveals a subset of evolutionarily old proteins for which a change in domain architecture coincides with an increased capability to infect the human host.

Another subject that had been discussed was the relationship between orthology and gene context. How function and gene clustering relate, and how gene clustering evolves across species, has been well studied in prokaryotes, but its significance in *Eukaryotes* remains understudied. A new method to detect evolutionary conserved gene clusters in eukaryotic genomes, EvolClust (Marcet-Houben and Gabaldón 2020), was presented, together with a first survey over 300 fungal genomes, which uncovered significant clustering and the functional and evolutionary patterns of fungal gene clusters (Marcet-Houben and Gabaldón 2019). Duplication events are often associated to chromosomal rearrangements and changes in the local genomic context, this property is exploited by a recently developed method that defines “primary orthologs” as those which never experienced a duplication event in their respective lineages that separate them (Gao and Miller 2020). Conserved gene order (or synteny) can also be useful to define orthology for genes that are poorly conserved at the sequence level, such as long-non coding RNAs, across species that nevertheless retain higher levels of synteny (Pegueroles et al. 2019).

Higher levels of biological organization and in particular the relationships between orthology and interaction networks have been another focus of the meeting discussions. The central premise is that orthologous proteins and genes which interact are likely a part of the same biological process. These interaction networks are often presented as graphs, whose nodes represent proteins and whose edges represent a functional association. Interaction networks encompass a broad variety of interaction types as well as methodologies to find those interactions (Huang et al. 2018). Several network interaction databases were represented, with talks discussing more precisely the latest improvements in STRING (Szklarczyk et al. 2019), FunCoup (Ogris et al. 2018), and KEGG (Kanehisa et al. 2019). These methods/databases focus on a variety of interaction types, including: protein–protein interactions, gene co-expression, protein-co-expression, genetic interaction profile similarities, shared transcription factor bindings, subcellular colocalization, domain interactions, cellular complex metabolic pathway, or signaling pathway comembership, and shared genomic contexts such as operons or gene neighborhoods.

Additionally, there are several methodologies to find evidence of interactions. In general, experimental screens and computational inferences are used to populate the networks. These methodologies can range from direct measurements of physical protein–protein interactions, which are then stored in curated online databases, to more indirect methods such as phylogenetic profile similarity, text-mining of published works, or machine learning algorithms to predict protein–protein interactions. STRING focuses on combining curated experimental databases, text-mining, and computational predictions (Szklarczyk et al. 2019), whereas FunCoup uses only high-throughput experimental data (Ogris et al. 2018).

Orthology is a fundamental tool to relate the interaction information from one species to another. Based on the premise that the interaction among proteins is evolutionarily conserved, the term “interologs” refers to “orthologous pairs of interacting proteins in different organisms” (Walhout et al. 2000; Yu et al. 2004). Simply put, if two proteins have been determined to interact in one species, one can infer that the two orthologs of those proteins in another species also interact. In FunCoup, functional associations from well-studied species are transferred to other organisms using orthologous relations from InParanoid (Sonnhammer and Östlund 2015), whereas STRING uses hierarchical orthologous relations from EggNOG (Huerta-Cepas et al. 2016). KEGG uses orthologous clusters, that is, computationally generated quasi-cliques of bidirectional best hits, a subset of which are manually curated to form KOs (22,937 clusters). Roughly, half of all 30 million proteins in KEGG have been assigned to KOs. From these orthology groups, KEGG pathway information from the curated molecular networks (KEGG pathway maps, BRITE hierarchies, and KEGG modules) can be assigned to orthologs belonging to the same KO group (Kanehisa et al. 2019).

Orthologs can also be used for not just relating the interaction network from one species to another, but also for comparing different gene interaction networks. For example, ManiNetCluster, a recent computational tool for comparing gene networks, can find functional links from multiple data sets (Nguyen et al. 2019). This could be used for relating gene expression networks based on different conditions or species.

### Seeking Quality in a Widened Genome Diversity

From the seminal recognition that molecular analysis could be used to graph biological diversity (Woese and Fox 1977; Woese et al. 1990), continued genome sequencing has provided more and more information into both “missing links” and previously unrecognized genomic diversity. Between the *Archaea* and the *Eukaryotes* for example, evidence has accumulated which seems to decrease the perceived taxonomic divide between these groups (Raymann et al. 2015; Spang et al. 2015; Imachi et al. 2020). Meanwhile, divergent organisms affiliated with both *Bacteria* and *Archaea* have been documented, revealing a previously unrecognized diversity of biology (Rinke et al. 2013; Brown et al. 2015; Adam et al. 2017). Said simply, our knowledge of life diversity has dramatically expanded (Hug et al. 2016; Parks et al. 2017), raising new challenges as to how to map orthology relationships to this diversity. Although single-copy gene trees revealed the overall contours of the tree of life (e.g., the analysis of 16sRNA, Woese and Fox 1977, or RpoB genes, Case et al. 2007), the phylogenomic approach involving concatenated sets of conserved (or highly conserved) orthologous proteins has aided in the acquisition of higher confidence species trees (Segata et al. 2013; Asnicar et al. 2020), which in turn has major implications for taxonomy (Parks et al. 2018).

Despite this critical need of establishing high-quality orthology prediction to resolve novel clades, semi-automated genome annotation remains the norm for most sequencing projects. *De facto*, the posterior establishment of high-quality gene models and high-quality reference proteomes is critical in the establishment of a reliable and stable orthology database, in particular when future functional annotations will propagate on the basis of these predictions. This remains true even for model species, in which erroneous annotations still lead to misinterpretation of in vivo experiments (Söllner et al. 2019). The following paragraphs report the QFO discussions related to building higher quality orthology models and the latest developments in this field. One point of recognition is that orthologs, paralogs, and the very idea of taxonomy changes over the wide diversity of organisms on Earth; because this diversity leads to unique challenges, different groups of organisms are discussed separately.

In *Eukaryotes*, orthology research has been intensive and many high quality orthology resources are now available, several of them having been created in the context of the QFO Consortium (Forslund et al. 2018). But for some gene families orthology predictions remain problematic and are often related to poor gene models. Incorrect eukaryotic gene models originate from diverse phenomena that were discussed during the conference. Although most mammalian protein sequences seem accurate, William R. Pearson showed how the process of building gene models can be sensitive to incorrect gap content and the selected similarity search programs (Pearson et al. 2017). Another common issue remains in the selection of incorrect isoforms, themselves related to potential errors of intron/exon predictions. This affects particularly gene tree reconstruction methods, where selected CDS might not be fully orthologous between species (different exon composition). In this regard, Aïda Ouangraoua discussed the concept of “CDS orthology,” where two homologous CDS are confirmed as orthologs after answering structural constraints in their splicing structure (e.g., number of introns, coding phases and lengths). This approach is implemented in SplicedFamAlign, a tool building splice-aware multiple sequence alignments (Jammali et al. 2019). Improving quality with better exon alignments was similarly a focus in the last release of the OrthoMaM database (Scornavacca et al. 2019). Its construction pipeline relies on the OMM_MACSE alignment pipeline (Ranwez et al. 2018), which limits frameshifts and splicing errors. Another issue was raised by Yuichiro Hara, who identified “elusive” genes in amniotes, for example, genes characterized by low phylogenetic conservation and lost in many taxa. He emphasized that making more complete reference proteomes is essential to distinguish genuine gene loss from information missing after an incomplete genome assembly or gene annotation (Hara et al. 2018). A similar point was raised in the talk of Shigehiro Kuraku, who showed that common approaches for gene space completeness assessment are insufficient to validate chromosome-scale assemblies built with Hi-C scaffolding (Kadota et al. 2020).

In *Bacteria* and *Archaea*, genes often show a lower structural complexity (shorter, single domain, no splicing, etc.) but for large-scale orthology inference, the preponderance of horizontally transferred genes (HTGs) is a challenge. The relation of “xenology,” introduced by Gray and Fitch (Gray 1984), initially described gene pairs related through such horizontal transfer. To date, detecting xenology from the gene pairs of orthologous groups typically produced by graph-based methods remains an open problem. Reconciliation algorithms accounting for gene transfers remain the only solutions for identifying potential xenologs (Altenhoff et al. 2019). For instance, PANTHER infers horizontal transfer events (Mi et al. 2016), and corresponding xenolog pairs, among a set of 142 fully sequenced genomes. Notably, HGTree is the first database dedicated to the detection of horizontal transfer and allows comparison of gene sets to species trees for thousands of bacterial and archaeal genomes (Jeong et al. 2016). More recently, a formal definition of xenolog classes has been proposed by Darby et al., 2017 and implemented in the reconciliation tool NOTUNG (Stolzer et al. 2012). Primary xenolog, sibling donor xenolog, sibling recipient xenolog and outgroup xenolog are the four proposed classes that reflect the events associated with the divergence of a xenologous gene pair and help to grasp the relative timing of the transfer and speciation events (Darby et al. 2017).

Another characteristic of *Bacteria* and *Archaea* species is that they often show a large genome diversity, that is characterized by a dichotomy between core genome and pan-genome (Tettelin et al. 2005). The core genome is associated with orthologous genes found in all strains at a given taxonomic level—for example at the species level—whereas the pan-genome represents the entire set of genes found in either strain of this given taxonomic level. Genes not belonging to the core genome are referred to as accessory genes. Whereas most of the core genes are likely to be vertically conserved, accessory genes can be acquired by HGT. The proportions of the core to accessory genes are different among species. Bacterial species characterized by sympatric lifestyle tend to have a smaller proportion of core genes than those of allopatric lifestyle (Golicz et al. 2020), suggesting these organisms acquire a substantial number of accessory genes from other organisms in the environment through HGT. On the other hand, as a consequence of HGT, genes with adaptive advantage in a specific environment tend to be shared among organisms in the same environment, and phylogenetic profiles of such genes show a characteristic sporadic rather than lineage-specific distribution. Such gene-sharing analysis (Dagan et al. 2008) is another approach to identify HGT through comprehensive orthology analysis. To integrate within-species and between-species comparisons, during the conference a comparative pan-genomic approach was introduced by Ikuo Uchiyama, who reused the idea of progressive orthology inference (Schreiber and Sonnhammer 2013) using taxonomic information in a bottom-up orthology inference from the strain level up to higher taxonomic levels. This approach is now implemented in the Microbial Genome Database for Comparative Analysis (MBGD) database (Uchiyama et al. 2019).

### Orthology and Viruses

An emerging application in orthology research is the particular case of virus genomes. Initially the meaningfulness of virus orthology was debated, but after decades of virus genome sequencing and with the expansion of metaviromics, comprehensive viral taxonomies are now well established (Eloe-Fadrosh 2019; Koonin et al. 2020). Undeniably, because there is likely no clear concept of species, virus genomes bring many new challenges to orthology inference. Moreover, there are no “universally” conserved genes in viruses and high evolutionary rates often limit comparative genomics to closely related genomes. Furthermore, xenology is complemented by analogy (e.g., host protein mimicking) and different virus families will show a tremendous variety of genome structures (from four genes in some *Geminiviridae* up to 2,500 genes in some *Pandoraviridae*, see Philippe et al. 2013). In addition, some viral families show specific evolutionary mechanisms such as reassortments (e.g., genome segments shuffling during coinfection), breaking the classical assumption of descendancy.

At the same time, the viral pan genome is often considered as the largest genetic reservoir on the planet. When a new virus of previously unknown lineage is discovered, most genes encoded in its genome have no homologs in extant databases. Likewise for metaviromic analysis, metagenomic analysis targeting subcellular fraction often results in 60–90% of reads with unknown origin; significantly higher proportion of those compared with the cellular fraction. Such reads or assembled contigs cannot be properly assigned to its biological origin—whether its host was archaeal, bacterial or eukaryal, and even often not sure whether it was cellular or viral origin—and often such reads are omitted from further analysis (Roux et al. 2012; Yoshida et al. 2013). These genes of unknown, but putatively of viral origin, are often referred to as the “biological dark matter.”

Compared with cellular organisms, the tremendous challenge of exploring the viral biosphere clearly shows that building virus orthology models is in its infancy and calls for specific research. To our knowledge, the first resource dedicated to virus orthology was the phage orthologous groups (POGs), a database of bacteriophages orthologs built from simple 3-way reciprocal BLAST matches (Kristensen et al. 2013). More recently, the Prokaryotic Virus Orthologous Groups (pVOGs) used a similar approach but enlarged the inference to nearly 3,000 bacterial or archaeal hosts (Grazziotin et al. 2017) and came with tools of functional annotations and phylogenetic profiling. Three generalist orthology databases (EggNOG, OrthoDB, and PhylomeDB) also offer viral orthologs but, similarly to POGs and pVOGs, their inference pipelines were initially developed for eukaryotic and prokaryotic genomes. It appears that the Vipr database (a general resource for virus genomics; Pickett et al. 2012), is the first resource using an orthology inference protocol developed specifically for viruses. A Domain-Architecture Aware Inference of Orthologs (DAIO), is used to classify viral proteins into “Strict Ortholog Groups” (SOGs), for example, groups where orthology relationships are confirmed via phylogenetic inference at low taxonomic levels and where domain architecture is conserved (Zmasek et al. 2019). This approach helped for the dissemination of functional annotation and naming conventions throughout the numerous viral families present in Vipr (Zmasek et al. 2019). A more recent resource is VOGDB (http://vogdb.org), a database proposing Virus Ortholog Groups (VOGs) inferred from phage and nonphage virus genomes. VOGDB pipeline uses a graph-based approach complemented by filters designed specifically for virus genomes structures (Kiening et al. 2019). Notably, it includes steps of polyprotein re-annotation and postclustering refinements based on HHalign-KBest (Yu et al. 2015), a hidden Markov model alignment method computing suboptimal alignments by using structural models, specifically designed for cases of low sequence identity (<35%). The database also provides tools developed for metagenomic applications such as identification of virus-specific markers or identification of orthologous groups which encode essential genes for viral lineages.

As shown by these recent examples, sequence homology alone appears insufficient for tracking distant viral lineages. Capsid genes have been previously discussed as an interesting criterion in distant comparisons (Krupovic and Bamford 2011). However, due to their polyphyletic origins and to their overwhelmingly rapid evolutionary rate, even these core genes can lose their sequence integrity. With the full genome sequence in hand identification of capsid genes can easily result in failure (Mochizuki et al. 2012). All together, this shows that sequence-based orthology has its usefulness in contexts where relatively recent viral divergences are studied. But it is an integrative approach, compiling both sequence and structure homology, that may be the key to decipher more ancient evolutionary relationships in the virosphere.

## Conclusion

The sixth QFO meeting/67th NIBB conference was an opportunity to gather the multi-faceted QFO community that now encompass people from a wide variety of biological domains—resource development, genome annotation, comparative genomics, evolution, biological networks—. It was also an opportunity to synthesize the challenges that result from the vast organismic and viral diversity that we can access today.

The latest developments in orthology inference have focused on scalability, community standards, and continuous developments for more integrated benchmarking and towards improved interoperability. Future efforts should focus on more reliable gene models and new means to share stable models and related large-scale computations that are common to many orthology resources. This is one of the core reasons for the consortium’s existence and the subject on which most progress has been done over the past years and will continue in the future.

Aside from the inference itself, efforts are directed to the multi-level components of orthology, from the gene-protein entity to its composing parts—exons, domains—and to its effects within larger biological structures—synteny, interaction networks—. Efforts are also directed toward democratizing uses of comparative genomics tools exploiting orthology and facilitating visualization of complex evolutionary patterns.

Finally, viruses appear as an emerging subject of orthology research. The genomic complexity of the panvirome, with its specific evolutionary mechanisms and fast evolutionary rates, is a major challenge for classical approaches and calls for dedicated tools. This crucial point is joining the many topics that will be examined by the QFO Consortium and will be the subject of future meetings.
